# Survival following a vertical free fall from 300 feet: The crucial role of body position to impact surface

**DOI:** 10.1186/1757-7241-19-63

**Published:** 2011-10-25

**Authors:** Sebastian Weckbach, Michael A Flierl, Michael Blei, Clay Cothren Burlew, Ernest E Moore, Philip F Stahel

**Affiliations:** 1 Department of Orthopaedic Surgery, Denver Health Medical Center, University of Colorado Denver, School of Medicine, 777 Bannock Street, Denver, CO 80204, USA; 2 Department of Surgery, Denver Health Medical Center, University of Colorado Denver, School of Medicine, 777 Bannock Street, Denver, CO 80204, USA

## Abstract

We report the case of a 28-year old rock climber who survived an "unsurvivable" injury consisting of a vertical free fall from 300 feet onto a solid rock surface. The trauma mechanism and injury kinetics are analyzed, with a particular focus on the relevance of body positioning to ground surface at the time of impact. The role of early patient transfer to a level 1 trauma center, and "damage control" management protocols for avoiding delayed morbidity and mortality in this critically injured patient are discussed.

## Introduction

Vertical deceleration injuries represent a significant cause of preventable deaths and long-term morbidity in survivors [1]. The amount of energy absorbed by the falling body is dependent on the fall height and the characteristics of the contact surface. For example, a fall onto concrete results in an instantaneous loss of speed, whereas falling onto a soft surface will allow for a more gradual deceleration over time [2]. In addition, the position of the body relative to the impact surface represents an important determinant of injury severity. The *American College of Surgeons' Committee on Trauma *(ACS-COT) defines a critical threshold for a fall height in adults as > 20 feet (6 meters), as part of the field triage decision scheme for transport to a designated trauma center [3]. A retrospective analysis of 101 patients who survived vertical deceleration injuries revealed an average fall height of 23 feet and 7 inches (7.2 meters), confirming the notion that survivable injuries occur below the critical threshold of a falling height around 20-25 feet [1]. A more recent study on 287 vertical fall victims revealed that falls from height of 8 stories (i.e. around 90-100 feet) and higher, are associated with a 100% mortality [4]. Thus, a vertical falling height of more than 100 feet is generally considered to constitute a "non-survivable" injury.

The present case report describes the rare survival of a 28-year old rock climber who survived a free fall from 300 feet onto a solid rock surface. This report emphasizes the crucial relevance of body positioning at the time of impact, and the importance of standardized institutional "damage control" management protocols for survival.

## Case report

A 28-year old woman was free climbing with her boyfriend near Gunnison, Colorado. Both were wearing a helmet and a harness for safety. The girl had 20 years of experience of rock climbing, being taught early tricks by her father at the age of 8 years. The ascent consisted of three pitches of 90-100 feet (ca. 30 m) each. The climbing distance was defined by the climbing rope which had been fixed at a defined length. The girl took the lead on the third pitch, to a total height of 300 feet (ca. 90 m). After securing the anchor at that height, the rope - which was lacking a security knot - slid through her harness. She then fell a total of 300 feet, with a first impact at 200 feet onto a flat rock surface, and a further fall for about 100 feet. Based on this falling height, the velocity at the time of impact is estimated around 75-80 mph. Her boyfriend witnessed the entire fall, climbed back down and provided first aid at the scene. The patient was awake and moaning, but not responsive to verbal or painful stimuli. She was intubated at the scene and transported to a local level IV trauma center, where she was resuscitated and transfused with 4 units of packed red blood cells (PRBC). Due to ongoing hypotension and transfusion requirements, a decision was made for transfer to our regional level 1 trauma center. On arrival, the patient was intubated and sedated. She was hypotensive, with systolic pressures in the 80s. She was successfully resuscitated with crystalloids and blood products, using a standardized institutional massive transfusion protocol with point-of-care thrombelastography-guided resuscitation [5,6]. The patient was managed according to the ATLS guidelines for initial assessment and management, and by our institutional "damage control" protocols, including the initial spanning external fixation of femur shaft fractures [7,8] and a proactive "spine damage control" approach [9].

The patient sustained the following combination of injuries:

• Blunt chest trauma with sternal fracture, bilateral hemo-/pneumothoraces, bilateral pulmonary contusions, right 1 and 2 rib fractures, left 9-11 rib fractures.

• Blunt abdominal trauma with grade 3 liver laceration, grade 2 splenic laceration, and a devascularized right kidney.

• Mild traumatic brain injury.

• Rotationally unstable flexion/distraction injury at T6 (AO/OTA type 52-C2.1) with traumatic spinal cord transsection and complete paraplegia ASIA grade A below T6.

• Unstable L1 burst/split fracture (AO/OTA type 53-A3.2).

• Unstable pelvic ring injury with bilateral SI-joint disruption, bilateral L5 transverse process fractures, bilateral pubic rami fractures, and left-side transalar/transforaminal Denis type 2 sacral fracture (Young-Burgess type LC-3, AO/OTA type 61-B3.3).

• Right femur shaft fracture (AO/OTA type 42-A3.2).

• Right type IIIA open talar body fracture (AO/OTA type 81-C3) and associated posterior facet calcaneus fracture (AO/OTA type 82-C2)

• Left comminuted joint-depression type calcaneus fracture (AO/OTA type 82-C3).

**Figure 1:**
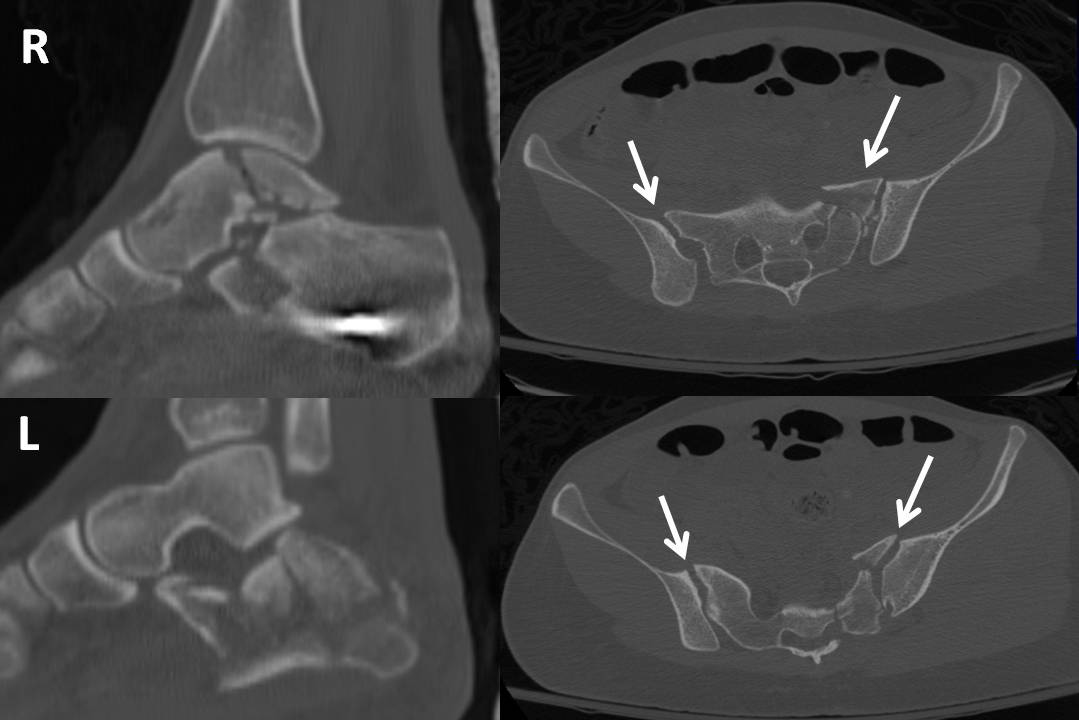
**Injury pattern of bilateral lower extremities and pelvic fracture on initial multislice CT scan**. The patient sustained a right-side open, comminuted talar body fracture, and a contralateral comminuted "joint-depression"-type calcaneus fracture, and a highly unstable pelvic ring injury with bilateral sacro-iliac joint disruptions (arrows).

The injury pattern of bilateral lower extremity fractures and of the pelvic ring injury are shown in Figure 1.

**Figure 2:**
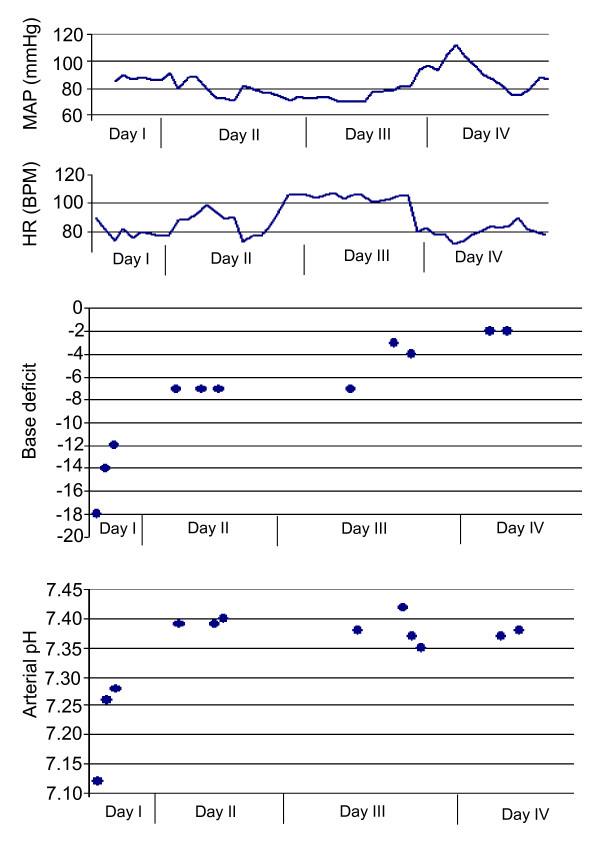
**Physiological response to resuscitation during the first 72 hours after trauma**. MAP, mean arterial pressure; HR, heart rate; BPM, beat per minute.

The chest trauma was managed by placement of bilateral chest tubes. The patient responded well to initial resuscitation and remained normotensive and well oxygenated, with a blood pressure of 115/80 mmHg, heart rate of 82/min, and 100% SO_2 _on 0.6 FiO_2_. She was taken to the operating room for "damage control orthopaedics" (DCO) procedure with unilateral spanning external fixation of the right femur fracture, surgical debridement of the open talar fracture with primary wound closure, and spanning external fixation of the right ankle in a delta-frame. The contralateral comminuted calcaneus fracture was placed in a well padded bulky Jones splint. The patient was then transferred to the surgical intensive care unit (SICU) for further resuscitation. The physiological response to resuscitation during the first 72 hours is depicted in Figure 2.

**Figure 3:**
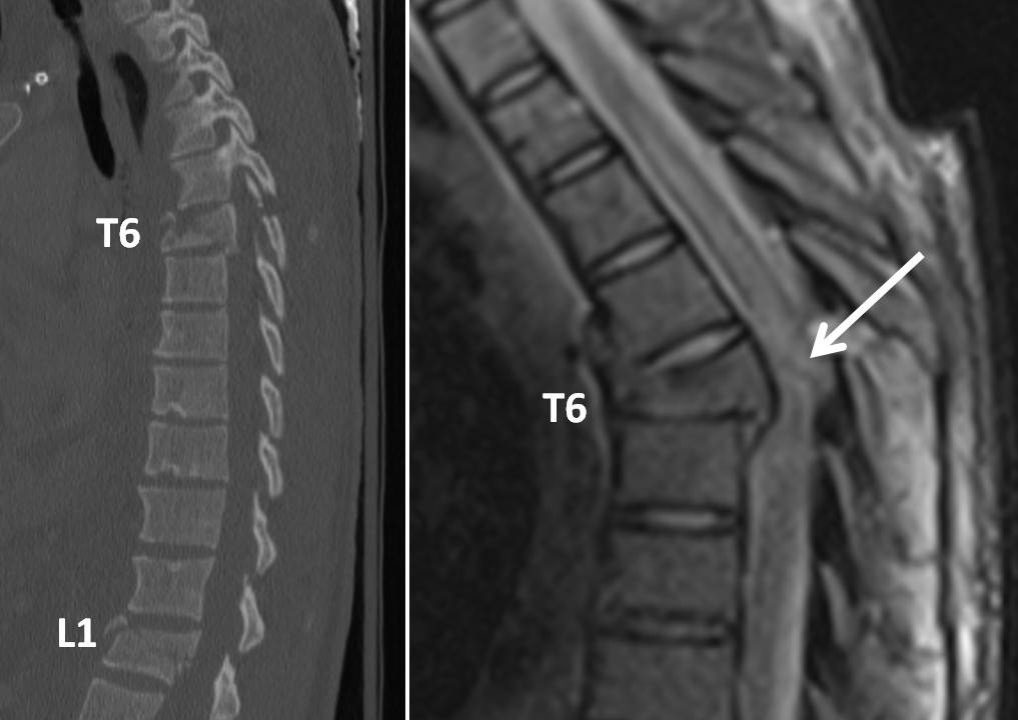
**Unstable spine injuries at T6 and L1 on initial multislice CT scan (left panel)**. The MRI of the T-spine (right panel) revealed a spinal cord transsection at the T6 injury level (arrow).

An MRI of her C-/T-/ and L-spine was obtained the next morning which documented a traumatic spinal cord transsection at the level of the rotationally unstable T6 flexion/distraction injury (Figure 3). She was taken the same day for preliminary spinal fixation as a "spine damage control" procedure [9]. This included a posterior spinal fusion from T4-T8 with laminectomy and spinal canal decompression at T6, as well as posterior spinal fusion T12-L2. The patient tolerated the procedure well and was brought back to the SICU in stable conditions. She was mobilized with physical and occupational therapy on day 1, and placed on low molecular weight heparin for DVT prophylaxis. The intraabdominal injuries were managed non-operatively.

On day 2, she was taken back to the operating room for stabilization of the pelvic ring injury using bilateral "triangular osteosynthesis" with lumbo-pelvic fixation from L4 to the ilium, and placement of bilateral 7.3 mm cannulated sacro-iliac screws through a safe surgical corridor [10]. On day 3, an IVC filter was placed due to the high risk constellation for a thromboembolic complication.

**Figure 4:**
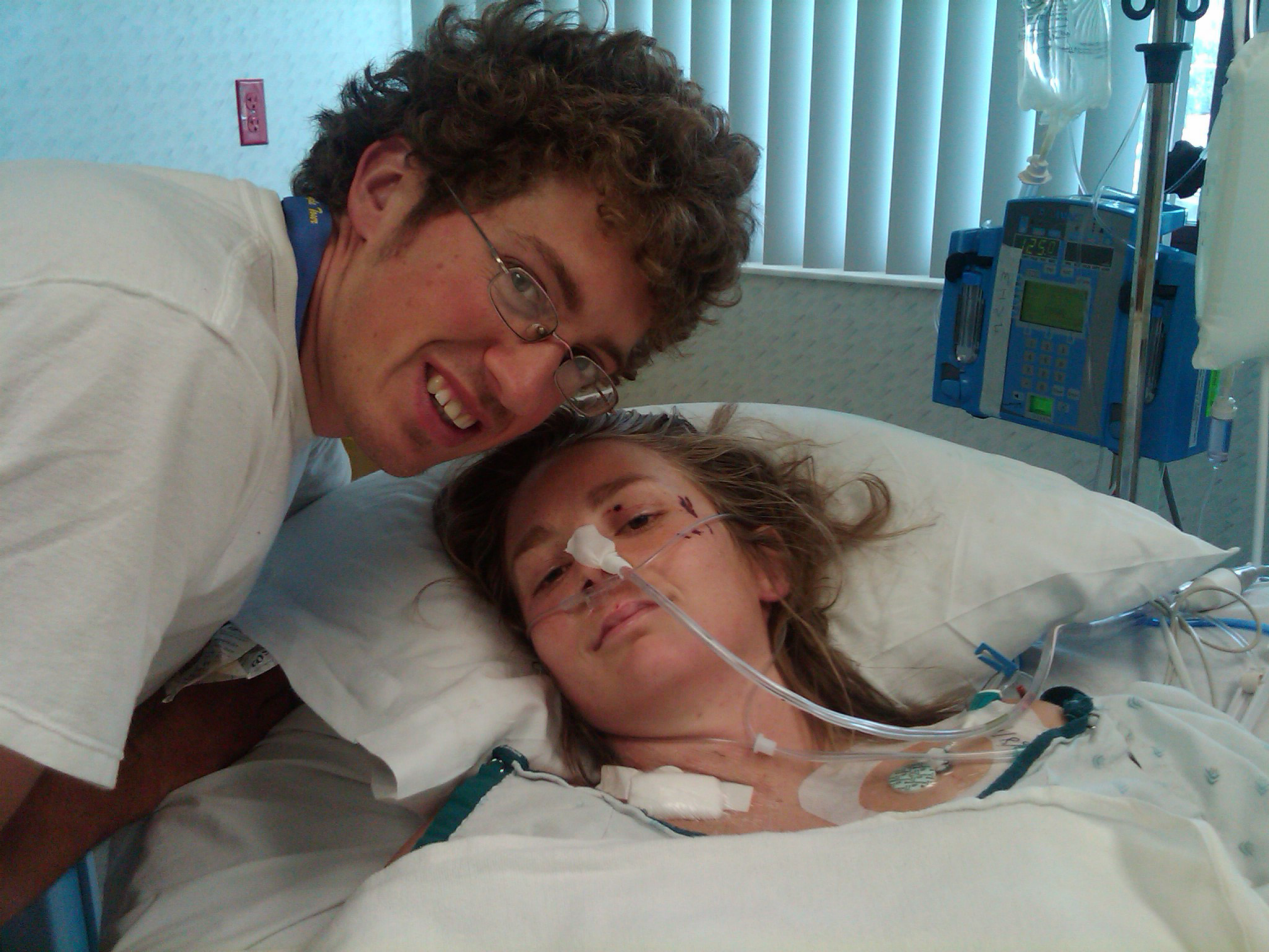
**The patient after successful extubation on hospital day 4, with her boyfriend who witnessed the free fall from 300 feet**.

**Figure 5:**
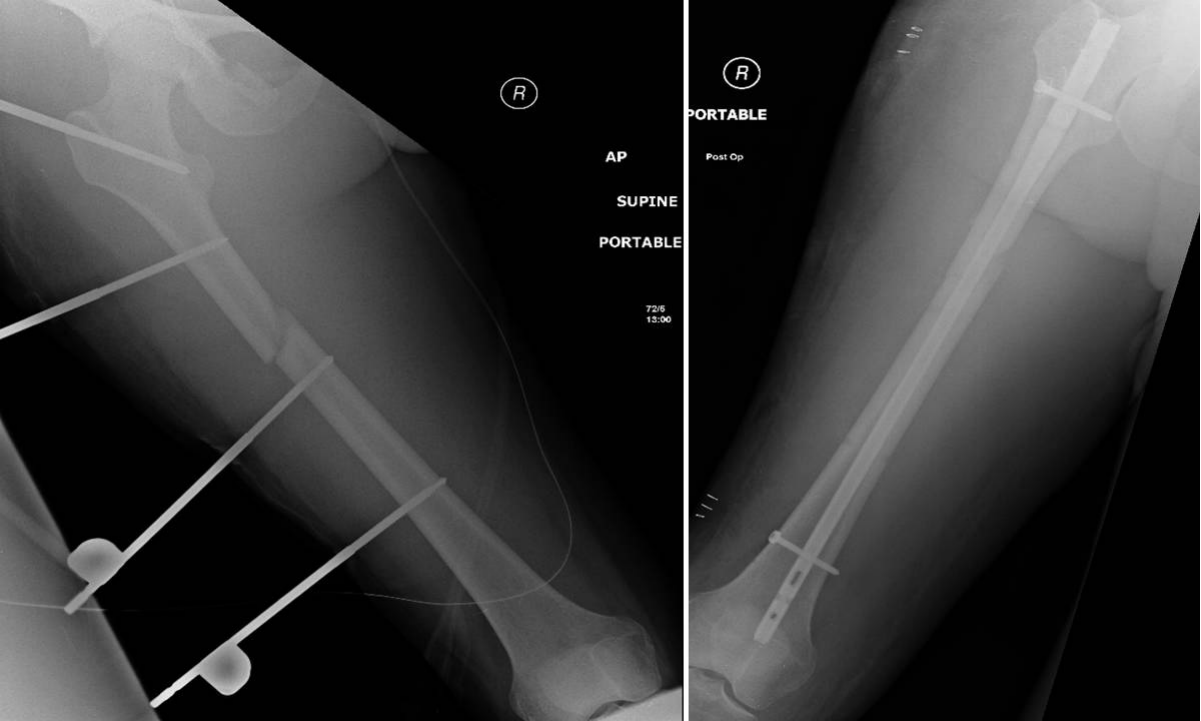
**Right femur shaft fracture managed by "damage control orthopaedics" with initial spanning external fixation (left panel) and delayed conversion to intramedullary nail fixation (right panel)**.

The patient recovered well from her injuries and from the "damage control" procedures. She was extubated on hospital day 4, and was successfully weaned to room air (Figure 4). She remained fully awake and alert, with a GCS of 15. She had a normal neurological function to bilateral upper extremities, but lack of sensory function below T6, and complete paraplegia to bilateral lower extremities. On day 5, she was taken back to the operating room for locked intramedullary nail fixation of the right femur shaft fracture (Figure 5), removal of the spanning external fixator, and cannulated lag screw fixation of her right talar body fracture.

**Figure 6:**
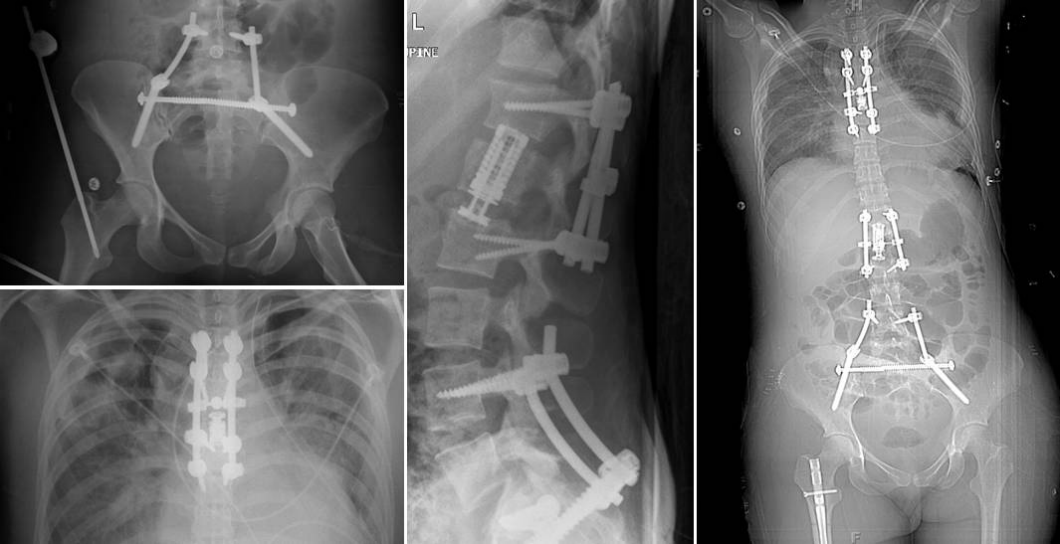
**Postoperative X-rays after stabilization of the pelvic ring injury with bilateral lumbo-pelvic/triangular osteosynthesis, and 360° fusion of the unstable T6 and L1 injuries**.

**Figure 7:**
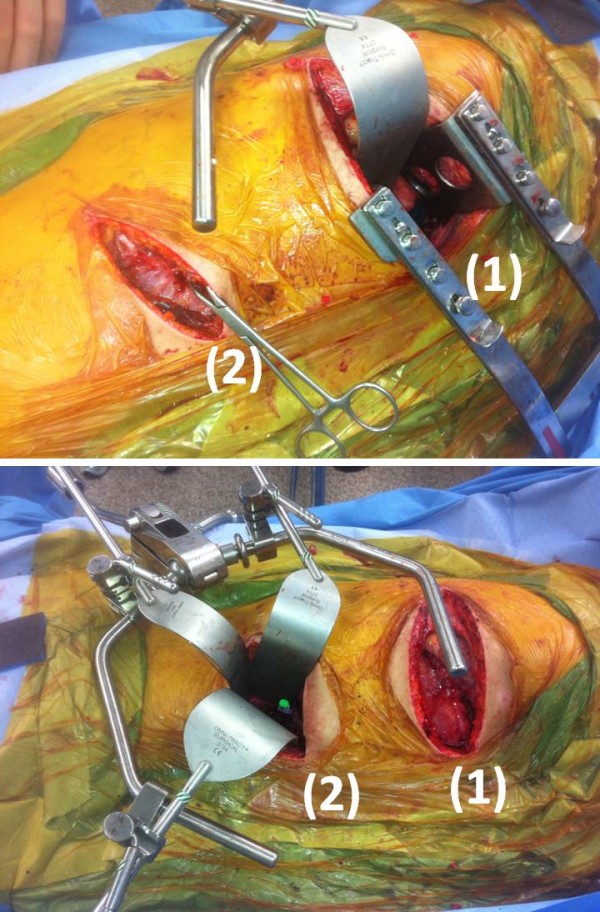
**Less-invasive two-cavity approach for anterior corpectomy, spinal canal decompression, and anterior spinal fusion of the unstable thoracic and lumbar spine fractures**. The T6 injury was managed through a small posterolateral thoracotomy (1), while the L1 fracture was addressed through a retroperitoneal approach along the 11^th ^rib (2). The less-invasive procedure was tolerated well by the patient, and allowed for early mobilization without restrictions.

The patient had an excellent recovery and was mobilized into a wheelchair with physical and occupational therapy. On day 13, she was taken back to the operating room for completion 360° fusion T5-T7 and T12-L2, with anterior corpectomy of T6 and L1 vertebral bodies, anterior spinal canal completion decompression, and placement of two titanium expandable cages and bone grafting (Figure 6). This procedure was performed through less-invasive left-side posterolateral approaches, including a transthoracic approach to T6 and a retroperitoneal approach to L1 (Figure 7). This less invasive technique was shown to be well tolerated by patients and allow early functional rehabilitation without restrictions [11-13].

**Figure 8:**
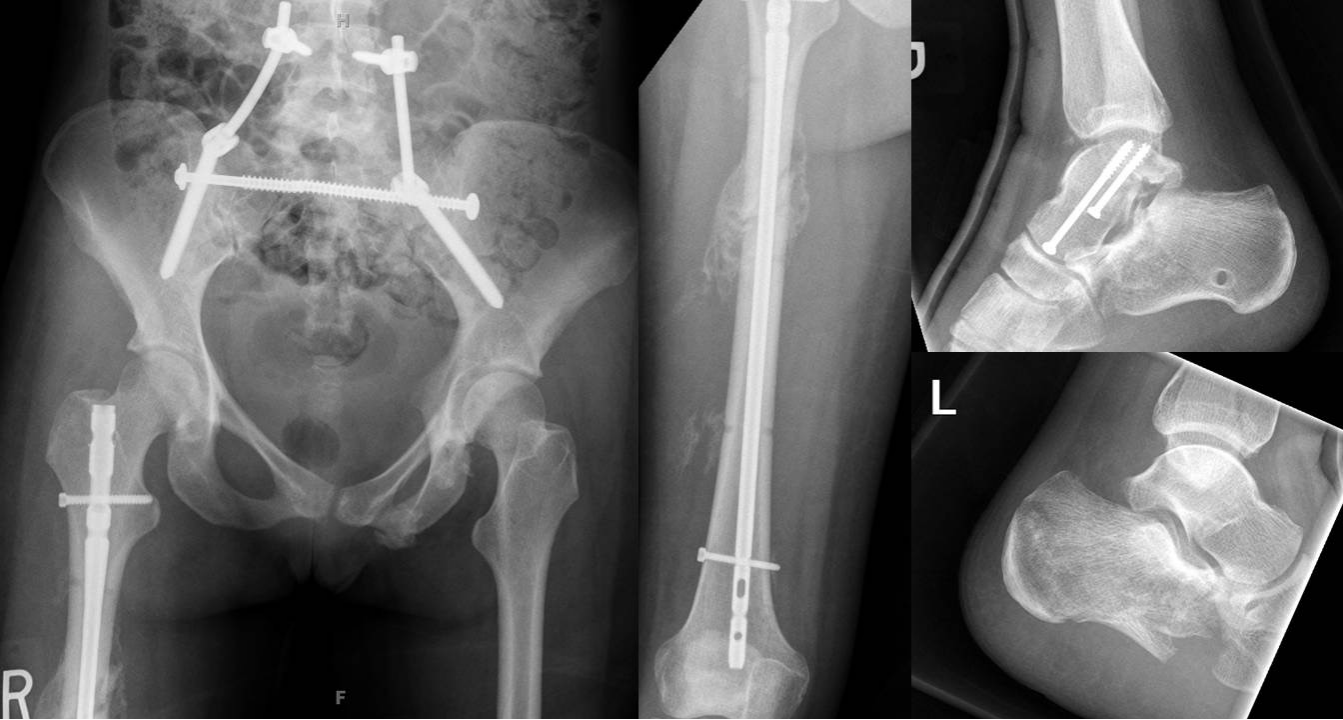
**Early fracture healing documented by X-rays obtained at 6 weeks post injury**.

The patient had an uneventful further recovery. All surgical wounds healed well, and there were no postoperative complications. At that time, X-rays of her multiple orthopaedic injuries were obtained, which showed early signs of uneventful fracture healing (Figure 8). She was transferred to our neurorehabilitation unit on hospital day 18. The patient remained flaccid below the level of lesion related to the T6 ASIA grade A complete spinal cord injury. She remained in spinal shock until approximately 6 weeks after trauma. She also showed some general processing and impaired short term memory deficits related to her mild traumatic brain injury. Nerve conduction studies confirmed the notion from MRI imaging, in that there was no secondary neurologic conus injury due to the L1 burst fracture which may have further complicated her bowel and bladder management. The applied spinal and pelvic fixation techniques facilitated her mobilization without adjunctive truncal bracing. The initial efforts for self-care and mobilization, however, were complicated by orthostatic hypotension, nausea and anxiety felt to be multi-factorial in etiology. The weight bearing precautions to the lower extremities were discontinued around 9 weeks post injury, based on progressive callus formation seen on follow-up X-rays (Figure 8). The patient quickly progressed to independent transfers. Her cognitive processing improved to essentially normal. The IVC filter was removed prior to discharge. She was transferred to her local community regional spinal cord rehabilitation center out-of-state at 2½ months after injury in excellent conditions, for completion of her neurorehabilitation program.

## Discussion

This is the first case report, to our knowledge, which documents survival from a free vertical fall of 300 feet onto a hard surface. The anecdotal threshold for sustaining critical injuries from a vertical fall has been defined by the *American College of Surgeons' Committee on Trauma *(ACS-COT) at >20 feet (6 meters) [3]. This threshold is corroborated by the published literature on survivors from accidental and suicidal free falls [1]. In general, a falling height of >100 feet is considered a "non-survivable" injury [4]. The height of 300 feet is ascertained by the fact that in "lead climbing", the climbing rope is fixed at a defined length, corresponding to 150 feet in the present case. The patient's boyfriend took the lead on the first pitch of 150 feet, where after she took over the lead on the next 150 feet. After securing the anchor at 300 feet height, the rope slid through her harness and she sustained an undamped vertical free fall onto a flat rock surface.

**Figure 9:**
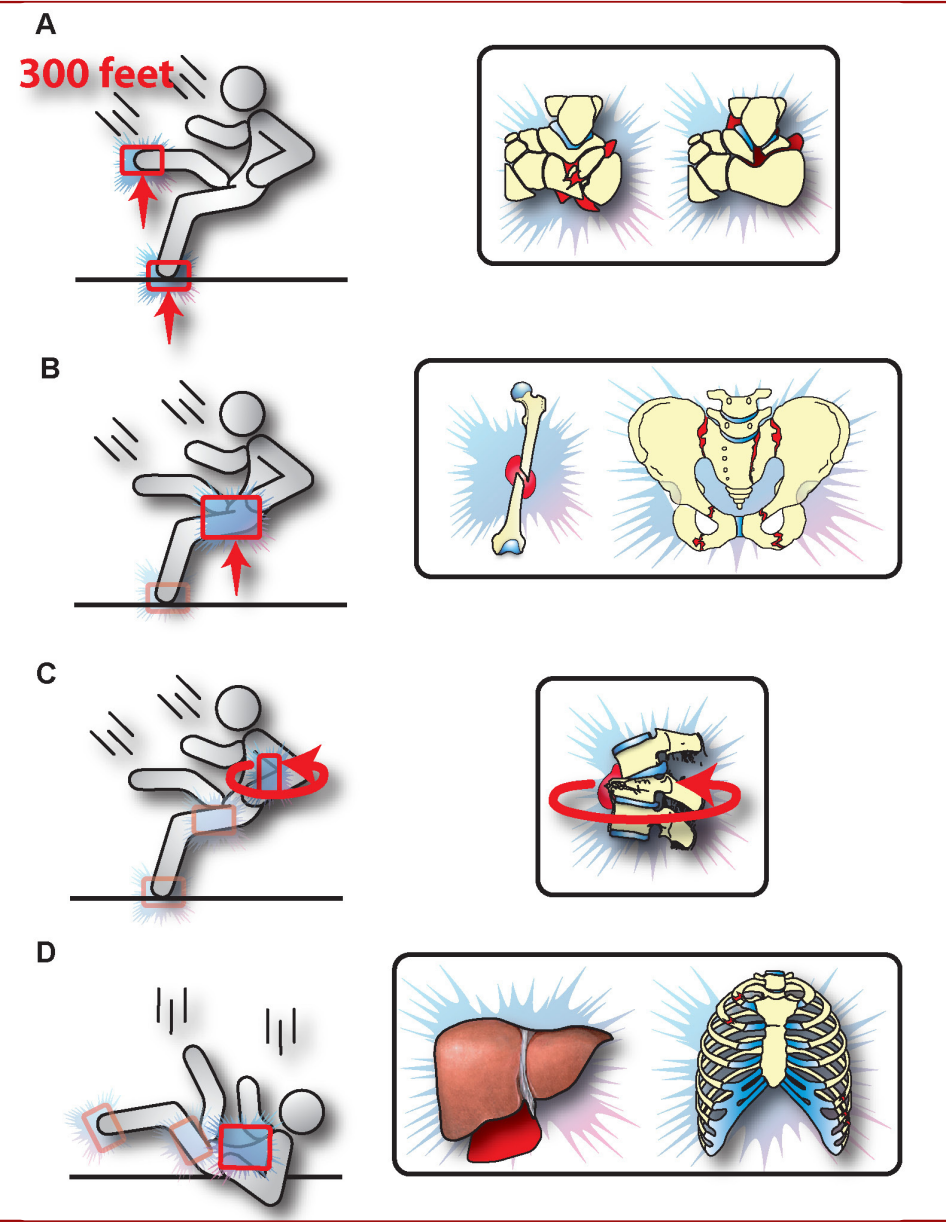
**Presumed trauma mechanism resulting from a 300 feet vertical fall in the present case**. Landing feet first is the likely root cause for survival in this 28-year old patient who sustained an injury mechanism generally classified as "non-survivable". Please refer to text for details.

Most falls from rock climbing result in simple sprains which affect ankle, elbow, and shoulder joints [14]. A retrospective analysis revealed that fractures of the spine and lower limbs represent the most frequent injury pattern in survivors from vertical falls from a height [1]. These findings concur with the notion presented in this case report, that a fall on both feet represents the "ideal" body to impact surface position with regard to survival from vertical falls. In contrast, brain injuries and cervical spine injuries resulting from a fall on the head represent the main cause for lethal outcomes after falls [15]. The patient's specific injury pattern is suggestive of a trauma mechanism by which the patient landed on both feet first, followed by a deceleration/twisting mechanism to her right femur and the thoracic and lumbar spine, ending in a fall on the back which induced the final deceleration forces leading to the intra-abdominal and thoracic injuries. As outlined by the presumed trauma mechanism depicted in the diagram in Figure 9, the patient landed feet first, leading to an energy transfer over a longer deceleration area from feet (**panel A**) to femur and pelvis (**panel B**) to a rotational flexion/distraction mechanism of the thoracic spine (**panel C**), followed by a fall on the back (**panel D**), which is associated with a distribution of the deceleration force over a larger surface area. Since the soft tissues and viscera decelerate slower than the skeleton, the final impact likely led to the chest trauma and intra-abdominal injuries to the parenchymal organs (**panel D**). This patient would likely not have survived the same injury mechanism, if she had landed head and neck first.

Furthermore, the rapid intubation, early resuscitation, and timely transfer to a qualified level 1 trauma center likely contributed to this patient's survival. It is striking to note that, despite the critical overall injury pattern, the patient did not sustain significant complications which may have been expected as the sequelae of the traumatic impact, including posttraumatic/postoperative infections, and the development of remote organ insults, including acute respiratory distress syndrome (ARDS) and multiple organ failure, which represent the main cause of late deaths in patients who survive the initial injury [16-18].

Likely, the application of standardized resuscitation strategies, in conjunction with thrombelastography-guided administration of blood products, and the limited exposure to the interventional burden by "damage control" strategies applied in the first few days after trauma, contributed to the survival of this patient [5-7,9,11,19,20].

The impact of falling height, quality of impact surface, and the position of the body to the impact surface on injury severity and outcome require further investigation in ex-vivo experimental and biomechanical studies.

## Competing interests

The author declares no competing interests with regard to this manuscript.

## Authors' contributions

PFS, EEM, and CCB designed this case report. PFS and SW drafted the first version of the manuscript. MAF contributed the graphic artwork in Figure 8. EEM and CCB managed the initial rescuscitation and performed all general surgery procedures. PFS performed all orthopaedic surgical procedures. MB was in charge of the neurorehabilitation of this patient. All authors contributed to the revised drafts of this manuscript and approved the final version of this paper.

## Written informed consent

Written informed consent for publication of this case report and of all radiological images and pictures was obtained from the patient by the senior author. She agreed to publish the case report including all figures shown in this paper. Written consent by the patient is available to the journal's Editor-in-Chief upon request.

## References

[B1] Richter D Hahn MP Ostermann PA Ekkernkamp A Muhr G Vertical deceleration injuries: a comparative study of the injury patterns of 101 patients after accidental and intentional high falls Injury 1996 27 9 655 659 10.1016/S0020-1383(96)00083-6 9039364

[B2] Bragg S Vertical deceleration: falls from height J Emerg Nurs 2007 33 4 377 378 10.1016/j.jen.2007.04.017 17643805

[B3] American College of Surgeons Committee on Trauma Resources for optimal care of the injured patient 2006 Chicago, IL: American College of Surgeons

[B4] Lapostolle F Gere C Borron SW Petrovic T Dallemagne F Beruben A Lapandry C Adnet F Prognostic factors in victims of falls from height Crit Care Med 2005 33 6 1239 1242 10.1097/01.CCM.0000164564.11989.C3 15942337

[B5] Stahel PF Moore EE Schreier SL Flierl MA Kashuk JL Transfusion strategies in postinjury coagulopathy Curr Opin Anaesthesiol 2009 22 2 289 298 10.1097/ACO.0b013e32832678ed 19390256

[B6] Kashuk JL Moore EE Sawyer M Le T Johnson J Biffl WL Barnett C Stahel PF Silliman CC Sauaia A Banerjee A Postinjury coagulopathy management: goal directed resuscitation via POC thrombelastography Ann Surg 2010 251 4 604 614 10.1097/SLA.0b013e3181d3599c 20224372

[B7] Stahel PF Smith WR Moore EE Current trends in resuscitation strategy for the multiply injured patient Injury 2009 40 Suppl 4 S27 35 19895950 10.1016/j.injury.2009.10.034

[B8] Flierl MA Stoneback JW Beauchamp KM Hak DJ Morgan SJ Smith WR Stahel PF Femur shaft fracture fixation in head-injured patients - when is the right time? J Orthop Trauma 2010 24 107 114 10.1097/BOT.0b013e3181b6bdfc 20101135

[B9] Stahel PF Flierl MA Moore EE Smith WR Beauchamp KM Dwyer A Advocating "spine damage control" as a safe and effective treatment modality for unstable thoracolumbar fractures in polytrauma patients: a hypothesis J Trauma Manag Outcomes 2009 3 6 10.1186/1752-2897-3-6 19432965 PMC2686673

[B10] Hasenboehler EA Stahel PF Williams A Smith WR Newman JT Symonds DL Morgan SJ Prevalence of sacral dysmorphia in a prospective trauma population: Implications for a "safe" surgical corridor for sacro-iliac screw placement Patient Saf Surg 2011 5 8 10.1186/1754-9493-5-8 21569232 PMC3105956

[B11] Haschtmann D Stahel PF Heyde CE Management of a multiple trauma patient with extensive instability of the lumbar spine as a result of a bilateral facet dislocation and multiple complete vertebral burst fractures J Trauma 2009 66 3 922 930 10.1097/01.ta.0000215415.87801.fc 18277288

[B12] Kossmann T Payne B Stahel PF Trentz O Traumatic paraplegia: surgical measures [German] Swiss Med Wkly 2000 130 816 828 10893753

[B13] Kossmann T Jacobi D Trentz O The use of a retractor system for open, minimal invasive reconstruction of the anterior column of the thoracic and lumbar spine Eur Spine J 2001 10 5 396 402 10.1007/s005860100330 11718193 PMC3611521

[B14] Gerdes EM Hafner JW Aldag JC Injury patterns and safety practices of rock climbers J Trauma 2006 61 6 1517 1525 10.1097/01.ta.0000209402.40864.b2 17159699

[B15] Stahel PF Heyde CE Flierl MA Wilkerson JA Wilkerson JA, Moore EE, Zafren K Head and neck injuries Medicine for Mountaneering 2010 Volume 6 Seattle, WA: The Mountaneers Books 86 95

[B16] Sauaia A Moore EE Johnson JL Ciesla DJ Biffl WL Banerjee A Validation of postinjury multiple organ failure scores Shock 2009 31 5 438 447 10.1097/SHK.0b013e31818ba4c6 18838942 PMC4324473

[B17] Keel M Trentz O Pathophysiology of polytrauma Injury 2005 36 6 691 709 10.1016/j.injury.2004.12.037 15910820

[B18] Stahel PF Smith WR Moore EE Role of biological modifiers regulating the immune response after trauma Injury 2007 38 12 1409 1422 10.1016/j.injury.2007.09.023 18048034

[B19] Burlew Cothren C Moore EE Smith WR Johnson JL Biffl WL Barnett CC Stahel PF Preperitoneal pelvic packing/external fixation with secondary angioembolization: optimal care for life-threatening hemorrhage from unstable pelvic fractures J Am Coll Surg 2011 212 628 637 10.1016/j.jamcollsurg.2010.12.020 21463801

[B20] Osborn PM Smith WR Moore EE Cothren CC Morgan SJ Williams AE Stahel PF Direct retroperitoneal pelvic packing vs. pelvic angiography: a comparison of two management protocols for hemodynamically unstable pelvic fractures Injury 2009 40 54 60 10.1016/j.injury.2008.08.038 19041967

